# Comparative validation of the IPAQ and the 7-Day PAR among women diagnosed with breast cancer

**DOI:** 10.1186/1479-5868-3-7

**Published:** 2006-03-31

**Authors:** Marilyn Johnson-Kozlow, James F Sallis, Elizabeth A Gilpin, Cheryl L Rock, John P Pierce

**Affiliations:** 1 San Diego State University Research Foundation, 9245 Sky Park Court, San Diego, CA 92123, USA; 2 San Diego State University, Department of Psychology, 3900 Fifth Avenue, San Diego, CA 92103, USA; 3 University of California, San Diego, Family and Preventive Medicine, 9500 Gilman Drive, La Jolla, CA 92093, USA

## Abstract

**Background:**

The criterion-related validity and measurement bias of the long form of the International Physical Activity Questionnaire (IPAQ) was compared to the 7-Day Physical Activity Recall (PAR).

**Methods:**

Participants were women who have been diagnosed with breast cancer and enrolled in the ongoing Women's Healthy Eating and Living Study. Women (*N *= 159, average age 57 years) wore an accelerometer for one week and then completed the IPAQ or the PAR.

**Results:**

The validity correlation of the PAR was significantly higher (*p *< 0.001) than the IPAQ (0.73 vs. 0.33, respectively). The PAR and IPAQ overestimated total physical activity by 13% vs. 247%, respectively. The PAR had better sensitivity (p = 0.14) and specificity (p < .01) than the IPAQ (100% vs. 71% and 84% vs. 59%, respectively) in predicting attainment of the ACSM physical activity guideline.

**Conclusion:**

The PAR was superior to the IPAQ in terms of validity, measurement bias, and screening statistics.

## Background

Many physical activity self-report measures have been developed [1-3] but few studies have compared the performance of different measures [4]. When new measures are developed, it is particularly useful to compare their psychometric properties to well-established measures in similar samples. Two self-report measures designed to assess overall physical activity examined in the present study were the 7-Day Physical Activity Recall (or PAR) [5,6] and the recently-introduced International Physical Activity Questionnaire (or IPAQ) [7].

The PAR [5] has been validated in a number of studies [3] and the purpose of the present study was to compare its criterion-related validity and measurement bias to the long form of the IPAQ. The two self-report instruments (PAR and IPAQ) were validated by comparing their physical activity estimates to those obtained from an objective physical activity accelerometer; the psychometric properties of the two self-report instruments were then compared. As both the PAR and IPAQ were designed to assess physical activity in all domains (occupation, recreation, travel, and housework) over the past 7 days and have similar administration durations, the comparison of the newly-developed IPAQ against the often-used PAR was warranted. Participants in the present study were women who were diagnosed with breast cancer and enrolled in a dietary intervention study, the Women's Healthy Eating and Living (WHEL) Study [8].

## Methods

### Procedures

Prior to any data collection, the institutional review board at the University of California, San Diego (UCSD), approved the study, and all study participants provided written informed consent. A convenience sample was recruited from the 531 study participants enrolled at the UCSD WHEL Study site who participated in a clinic visit between June 2001 and July 2002. At that time, demographic data, height, and weight were made available from the WHEL Study, and body mass index was calculated as weight (kg)/height (m)^2^.

During the first half of the recruitment period, participants were administered the International Physical Activity Questionnaire [7] and during the second half of the recruitment period, women were administered the 7-Day Physical Activity Recall [5,6]. Site coordinators initially agreed to allow the IPAQ to be administered to study participants and then at a later time, after the IPAQ had been administered, allowed the administration of the PAR. For this reason, it was not possible to randomly assign the women to receive either the IPAQ or the PAR. To minimize participant burden of the women, who were required to complete a significant number of questionnaires and measurements as part of the WHEL Study, participants completed either the IPAQ or the PAR.

Women were asked at the clinic visit by WHEL Study staff whether they wanted to participate in the study. Following their clinic visit, interested WHEL Study participants met with the first author to review study procedures. During that meeting, the telephone-administered IPAQ or PAR was scheduled and participants were fitted with an accelerometer. Participants also received informational handouts describing the study protocol, the physical activity interview (either the PAR or the IPAQ, as assigned), and a calendar showing the 7 days to wear the monitor. The study handout introduced the purpose of the study, details about the accelerometer, when and where to wear the monitor, and the schedule for wearing the monitor. The handout describing the physical activity interview, either the PAR or the IPAQ, described what the interview covered, what kinds of questions would be asked, and when it would take place. A copy of the handouts and all study materials are available for review [9].

After wearing the accelerometer for a week, participants completed either the PAR or the IPAQ and returned the accelerometer in the mail. Both the PAR and the IPAQ interviews assessed physical activity over the past 7 days that the women wore the accelerometer. Accelerometer data were downloaded and analyzed, and a report of each woman's physical activity was distributed as an incentive for participation.

### Measurement instruments

#### Actigraph accelerometer

The accelerometer, Actigraph Model 7164 (Manufacturing Technology Inc., Fort Walton Beach, FL), was placed in a pouch on a belt around the waist midway between the navel and the right side of the body. Participants were instructed to wear the accelerometer from the time they awoke in the morning until they retired at the end of the day. They were to remove the accelerometer during any water-based activities, such as swimming or bathing, as the Actigraph was not waterproof.

The accelerometer was programmed to record physical activity in the default 1-minute intervals. The computer programs which accompany the Actigraph yielded time spent in moderate and vigorous physical activity. Moderate physical activity corresponded to 3.0 to 5.9 METs, where 1 MET, or metabolic equivalent, is defined as energy expended in comparison to resting metabolism during quiet sitting. Vigorous physical activity corresponded to 6.0 METs or more, based on published calibration data [10]. Total physical activity was the sum of time spent in moderate and vigorous physical activity. Consistent with previous research methodology [7] time spent in sedentary behavior was defined as an activity count of less than 100.

#### 7-Day Physical Activity Recall (PAR)

The PAR was administered by the first author previously trained to criterion in the standard protocol [11]. Briefly, this approach used cues and prompts to help participants recall significant events over the previous 7 days, starting with the most recent day and working backward in time. The interviewer then used these events to frame their recall of physical activity. Participants classified each physical activity as moderate, hard, or very hard by using walking at a brisk pace as the standard for moderate activity and running for very hard physical activity. Complete instructions for PAR administration have been published [11].

#### International physical activity questionnaire

The long form of the IPAQ was administered by telephone, requiring about 20 minutes to complete. Unlike the PAR, which did not use a predetermined set of items, the interviewer read a standard set of questions to the respondent and then recorded the answers. The IPAQ assessed moderate and vigorous physical activity in four life domains: job-related work done outside the home (paid jobs, farming, volunteer work, and course work), house and yard work (outside and inside the home), recreation, and transportation (to locations such as work by bicycle or automobile, train, or other motor vehicle) among young and middle-aged adults [7]. It included a separate measurement of time spent sitting at a desk, visiting friends, reading, or watching television. Walking was assessed as part of occupation, transportation, and recreational activities but its intensity was not measured. Moderate physical activity was described as activities that made you breathe somewhat harder than normal; vigorous physical activity was described as activities that made you breathe much harder than normal. Physical activity was measured in a frequency-by-duration format on a per week basis. The long form of the IPAQ instrument is available online at .

Total time spent in physical activity during the past 7 days was obtained by multiplying the number of days the activity was done by the amount of time spent in each activity and then summed according to the intensity of the physical activity. Time spent in moderate activity included activities done as part of occupation, housework (including yard work, house maintenance and caring for family), recreation (including sports and leisure-time activities), bicycling (travel and recreational) and all walking. Vigorous activities included those performed as part of occupation, housework, and recreation. Total physical activity was the sum of time spent in moderate and vigorous physical activities. Self-reported time spent sitting as assessed by the IPAQ was the sum of the time spent sitting on a typical week day (for example, sitting at a desk, watching television, and reading) and time spent in a vehicle, such as a automobile, bus, or train, for the purpose of transportation from place to place.

### Statistical analysis

All analyses were conducted with SPSS Version 10 (SPSS, Inc., Chicago, IL, 1999). A two-tailed hypothesis was used for all statistical analyses with an alpha level set at 0.05. Measurement bias was defined as the difference in self-reported minus accelerometer minutes of physical activity and indicated how well the self-report measures assessed the absolute amount of physical activity performed by the women. The normality of the frequency distributions of all continuous variables was evaluated by the Kolmogorov-Smirnov statistic; only age was normally distributed. Accordingly, the Wilcoxon signed-rank test was used to compare the differences between the self-report and accelerometer scores. Bland-Altman plots [12] and intraclass correlations indicated the agreement of IPAQ and PAR scores with accelerometer scores. Criterion-related validity of the two self-report physical activity instruments was assessed by Spearman rank-order correlation. The statistical difference in the correlations between the PAR and IPAQ was based on an independent samples statistical test using a z-score transformation. The prediction of the proportion of women meeting the American College of Sports Medicine (ACSM) physical activity guideline [13] was evaluated by McNemar's Test for Correlated Proportions. Meeting the ACSM guideline was defined as engaging in 30 or more minutes per day of at least moderate physical activity on 5 or more days of the week [13]. As the guideline is based on a 7-day week, these analyses were limited to women who wore the accelerometer for 7 full days. The chi-square test was used to evaluate the difference in specificity and sensitivity between the PAR and IPAQ. Cohen's kappa was computed to indicate the magnitude of agreement between the self-report and accelerometer-based classifications; percent agreement in meeting the guideline was also reported. The number of hours spent sedentary per day was obtained from the accelerometer and compared to the IPAQ self-reported total time spent sitting. Outliers, defined at 3 standard deviations about the mean, were identified, resulting in two outliers each on the IPAQ and accelerometer; no outliers were identified on the PAR. However their elimination did not materially modify the results and the presence of the outlying values on the Bland-Altman plots allowed for the full investigation of measurement bias. Therefore, outliers were not eliminated from the analyses.

## Results

### Recruitment and sample exclusions

WHEL Study clinic staff recruited 180 women to participate in the study. Two of the 180 women did not complete any part of the study. Both of these women were in the IPAQ-administered group and did not participate in the study due to illness. Of the 178 women who participated in the study, 104 were in the IPAQ-administration group and 74 were in the PAR-administration group. Of the 104 women who completed the IPAQ, two were eliminated due to an accelerometer malfunction, so 102 had accelerometer data available for analyses. As determined by the IPAQ interview, six women in the IPAQ-administered group had more than half of their physical activity, such as water-based activity, not measured by the monitor. Therefore, a total of 96 of the 104 women in the IPAQ-administration group were included in statistical analyses.

Of the 74 women who were scheduled to complete the PAR, one was excluded due to an accelerometer malfunction, two did not complete the PAR, and eight had more than half of their physical activity not measured by the monitor. The final sample size for the PAR-administered group was 63. The total sample of 159 was comprised of those with data available on either the IPAQ (*N *= 96) or PAR (*N *= 63). Women wore the accelerometer on average for 15.2 hours per day (SD = 1.5); this was estimated to be 98% of their monitoring week.

Age ranged from approximately 35 to 77 yr at the time of recruitment into the study, with an average age of 56.6 yr. Body mass index varied from approximately 18 to 46, with an average of 26.9 kg·m^-2^. Most (91%) women were non-Hispanic white with at least a college education (56%). At the time of recruitment into the present investigation, there were no differences (*p *> 0.05) in time spent in total physical activity (based on the accelerometer), age, body mass index, and the proportion non-Hispanic white or college-educated between PAR- and IPAQ-administered groups (see Table 1).

**Table 1:** Differences between IPAQ- and PAR-administered groups (N = 159)

	IPAQ (*N *= 96)	PAR (*N *= 63)	IPAQ vs. PAR *p*
	Mean (SD)		
Total Physical Activity (min·wk^-1^)	181.2 (159.9)	164.7 (105.8)	0.89
Age (yr)	57.5 (9.1)	55.4 (9.1)	0.17
Body Mass Index (kg·m^-2^)	27.0 (5.6)	26.9 (5.8)	0.92
	Percent (%)		
Non-Hispanic white vs. other	93.8	87.3	0.26
College vs. non-college education	56.3	55.6	0.93

The *p*-values refer to tests of the difference in means (independent t-tests or the Wilcoxon rank sum test) or percents (Pearson chi-square) between groups administered either the International Physical Activity Questionnaire (IPAQ) or the 7-Day Physical Activity Recall (PAR).

### Measurement bias

Table 2 reports descriptive statistics of time spent in total physical activity in min/wk based on the accelerometer, IPAQ, and PAR. The minimum scores on all of the measures were very similar. The maximum total physical activity score for the IPAQ was approximately three to four times greater than obtained on the PAR or accelerometer. The mean total physical activity on the IPAQ was approximately 2.5 times and the median was 2.0 times that of the other measures.

**Table 2:** Accelerometer descriptive statistics and difference with self-report measures in minutes/week (N = 159)

	Descriptive Statistics			Self-Report Minus Accelerometer					
	Accelerometer (N = 159)			7-Day Physical Activity Recall (N = 63)			International Physical Activity Questionnaire (N = 96)		
	Mean (SD)	Median	Range	Mean_dif _(SD_dif_)	Median_dif_	Range_dif_	Mean_dif _(SD_dif_)	Median_dif_	Range_dif_
	
Moderate	162 (122)	143	4 to 771	+17 (102)	-1	-203 to 240	+225 (367)***	+108	-332 to 1447
Vigorous	13 (43)	0	0 to 367	+5 (32)	0	-97 to 162	+31 (130)**	0	-166 to 980
Total	175 (141)	145	4 to 826	+22 (104)	-2	-188 to 240	+257 (408)***	+130	-345 to 1791

**p < 0.01 ***p < 0.001; *p*-values indicate a difference between self-report and accelerometer scores based on a two-sided Wilcoxon signed-rank test.

The PAR overestimated total physical activity by 22 minutes per week on average (or 13%); this was not significantly different from zero (see Table 2). This was based on the combined overestimation of 17 minutes per week of moderate (11%) and 5 minutes per week (41%) of vigorous physical activity compared to the accelerometer.

Total physical activity as reported on the IPAQ was significantly higher compared to the accelerometer (*p *< 0.001). The IPAQ averaged 257 minutes (median = 130 min/wk) more than the accelerometer (or 247%). The IPAQ significantly overestimated moderate physical activity by about 225 minutes per week or 239% (*p *< 0.001) (median = 108 min/wk) and significantly overestimated vigorous physical activity by about 31 minutes per week or 342% (*p *< 0.001) (median = 0 min/wk).

**Figure 1:**
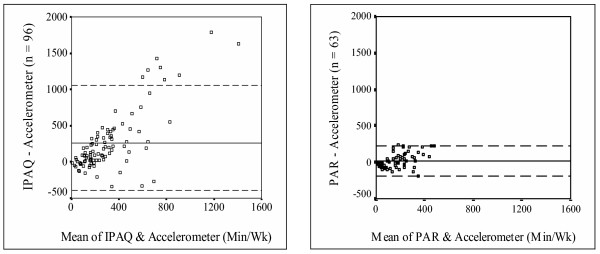
**Bland-Altman plots of IPAQ and 7-Day PAR**. Bland-Altman plots of self-reported total physical activity for the International Physical Activity Questionnaire (IPAQ) and the 7-Day Physical Activity Recall (PAR). The solid line is the average of the mean difference between the IPAQ and PAR; the dashed line is the 95% *CI *around the mean differences

Figure 1 shows the Bland-Altman plots for the two self-report measures of total physical activity. In order to assist in interpretation, a standardized scale was used for both measures. The solid line on the plot indicates the mean of the difference between self-report and accelerometer score. These values are the same as those reported in Table 2: +257 and +22 min· wk^-1 ^for the IPAQ and PAR, respectively. The dashed lines indicate the 95% confidence interval (*CI*) around the mean of the difference scores between self-report and accelerometer-based total physical activity. Consistent with measurement bias results, the 95% *CI *was larger around the IPAQ measure in comparison to the PAR. The PAR had fewer scores outside the 95% *CI*, indicating fewer extreme outliers on the PAR compared to the IPAQ. The magnitude of the overestimation on the IPAQ was associated with the amount of physical activity measured by the accelerometer.

### Criterion-related validity

The validity correlation coefficient of total physical activity scores for the PAR was 0.73 and for the IPAQ was 0.33. The correlations were significantly different from each other (*p *< 0.001) and both were significantly different from zero (*p *< 0.001). The statistical power for the test evaluating the difference between the correlations was greater than 0.80. The intraclass correlation with the accelerometer was 0.68 and 0.04 for the PAR and IPAQ, respectively.

### Sensitivity, specificity and related statistics

Table 3 shows the number of women identified as meeting or not meeting the physical activity guideline as determined by accelerometer, IPAQ (*N *= 88) and PAR (*N *= 58), based on those 146 women who wore the accelerometer for the full 7-day monitoring period. According to the accelerometer, 25 of the 146 women, or 17%, met the ACSM physical activity guideline. The PAR categorized 28% (or 16 of 58 women) as meeting the guideline; the comparable value was 47% (or 41 of 88 women) for the IPAQ. McNemar's Test for Correlated Proportions indicated that both of the self-report scores resulted in a significantly greater proportion of women meeting the guideline than the accelerometer (*p *< 0.01). McNemar's Test for Correlated Proportions had better than 80% power to detect differences among the observed correlated proportions.

**Table 3:** Number of participants meeting physical activity guideline by self-report vs. accelerometer (*N *= 146)

	Accelerometer				Agreement	
Self-Report	Met Guideline	Did Not Meet	Sensitivity	Specificity	%	Kappa
IPAQ (*N *= 88)*						
Met guideline	12	29	0.71	0.59	61	0.21
Did not meet	5	42				
PAR (*N *= 58)*						
Met guideline	8	8	1.00	0.84	86	0.59
Did not meet	0	42				

*The percent meeting the American College of Sports Medicine guideline according to the accelerometer significantly differed from both self-report measures at *p *< 0.01 based on a two-sided test of McNemar's Test for Correlated Proportions.

The sensitivity for the IPAQ, as shown in Table 3, was 71% (or 12 out of 17 were accurately identified as meeting the ACSM guideline) and for the PAR was 100% (or 8 out of 8 were accurately identified as meeting the guideline); these percentages were not statistically different (*p *= 0.140). The specificity for the PAR was significantly higher (*p *= 0.007) at 84% (or 42 out of 50) than that of the IPAQ at 59% (or 42 out of 71). The specificities indicate that the IPAQ more often misidentified women as meeting the guideline compared to the PAR, 41% vs. 16%, respectively.

There was 61% and 86% agreement between accelerometer and IPAQ and PAR classifications, respectively. Based on Cohen's kappa, this resulted in poor agreement for the IPAQ (κ = 0.21) and fair to good agreement for the PAR (κ = 0.59) using published interpretation guidelines [14].

### Time spent sitting

The average time spent sitting was 6.4 (SD = 3.5) and 8.3 (*SD *= 1.7) hours per day for the IPAQ and accelerometer, respectively. This difference was significant (*p *< 0.001) based on the Wilcoxon signed-rank test. The Spearman rank-order correlation between self-report and accelerometer-based time spent sitting scores was 0.49.

## Discussion

The present investigation compared the psychometric properties of the IPAQ with those of the more widely-used PAR measure of physical activity. Compared to the data collected via accelerometer, the validity correlation coefficient of the PAR [3,15] was substantially higher (0.73 vs. 0.33, respectively) than that observed with the IPAQ [7]. Compared to the accelerometer, the PAR overestimated total physical activity by a nonsignificant 13%. In comparison, the IPAQ overestimated it by 247%. The PAR also had greater sensitivity (100%) and specificity (84%) than the IPAQ (71% and 59%, respectively). On all comparisons, the PAR was superior to the IPAQ.

Disparity in their psychometric properties may be due to differences in the approaches the two measures used to assess physical activity. In comparison with the standardized set of IPAQ items, the PAR interview method was comparable to cognitive interviewing techniques. The cognitive interview uses a guided memory technique in which interviewees are encouraged to visualize the location of a past event and to recall event-specific details. This technique has been shown to improve recalls of dietary behaviors [16,17] and physical activity [18-20]. In an application of this technique, Johnson and colleagues [19] used simple printed instructions to list various types of walking activities, such as hiking and walking the dog, which resulted in significantly higher validity correlation coefficients and lower measurement bias on a paper-and-pencil survey of walking.

Cognitive processing research indicates that recall of behavior which is frequent but irregular, as is true of many, if not most, physical activities, is aided by providing cues which elicit memories about when, where, and how the behavior occurred [21]. Alternatively, physical activity that is structured or occurs infrequently may be accurately assessed by either a rate-based heuristic or enumeration strategy [18]. This may be one reason why the PAR resulted in significantly greater specificity compared to the IPAQ. The PAR was possibly more accurate in eliciting recall of the more commonly performed incidental physical activities in comparison to the IPAQ. The multiple-choice format of the latter would be expected to provide a more accurate assessment of routine or structured physical activities.

The PAR resulted in a nonsignificant overestimation of total physical activity by about 22 minutes per week (13%), of which 17 minutes was moderate physical activity. However, this may not necessarily reflect measurement bias of the PAR. Leenders and colleagues [22] have demonstrated that energy expenditure based on the PAR was not significantly different from that obtained by doubly labeled water method but that the Actigraph accelerometer underestimated energy expenditure. The uniaxial accelerometer is known to not measure all physical activity [23]. It underestimates physical activity conducted primarily above the waist, such as sweeping or carrying a load, or activity done on an incline, such as running up a hill, and does not measure water-based activity at all.

The IPAQ resulted in significant over-estimation of moderate physical activity in part because it included walking of any intensity, as has been reported previously [24]. Including all walking in the moderate physical activity score was based on previous research [7] that indicated additional questions concerning intensity or pace of walking did not significantly affect relative validity, which is based on the correlation coefficient. It is unlikely that the inclusion of walking pace would affect the relative validity, but it would likely have a substantial impact on the absolute validity, or the absolute amount of physical activity reported. In the present study, based on women with an average age of 57, the pace of a significant proportion of the walking may have been of light intensity.

The IPAQ assessed physical activity in four life domains, including occupational, transportation-related, house and yard work, and recreational and sports activities. Many of these activities may be under-estimated by the accelerometer. Vacuuming, sweeping, lifting, digging, climbing stairs, carrying loads, raking, washing windows, and swimming are exemplars listed on the IPAQ. Each is moderate in intensity, but would be inaccurately classified as light by the accelerometer. The more kinds of physical activity assessed by the IPAQ, the more likely it was to result in over-estimates of physical activity compared to the accelerometer. The sensitivity and specificity of identifying women meeting or not meeting the ACSM physical activity guideline [13] favored the PAR over the IPAQ. This was somewhat predictable, because the recommendation was based primarily on leisure activity while the IPAQ assessed four domains of physical activity.

While validity studies of these measures have been previously conducted in independent samples in separate studies, the present investigation compared the validity of the IPAQ and PAR in highly similar samples from a common group of study participants. Nevertheless, the total IPAQ validity correlation coefficient obtained in this study was similar to that obtained in the original IPAQ validation study [7]. Across 14 validation sites worldwide, a median rank-order correlation of about 0.30 was obtained comparing the accelerometer with MET-weighted minutes of total physical activity [7]. Consistent with the present study, the value of the median rank-order correlation was smaller than the total physical activity validity correlations obtained for the PAR in three previous studies, which were 0.50, 0.53, and 0.41, using an accelerometer as the criterion [3,15].

The present study is also consistent with previous research on the over-reporting of total physical activity on the IPAQ [7]. In a sample of 304 male and female volunteers in Sweden, Finland, England, and the United States, total physical activity was 1,085 min· wk^-1 ^using the self-administered version of the IPAQ long form [7]. This was 773 min· wk^-1 ^on average greater than that obtained from the Actigraph accelerometer. Thus, the IPAQ total time was about 3.5 times higher than that obtained from the accelerometer; the comparable amount in the current study was 2.5 times higher. In the previous study [7], time spent sitting was well-estimated by the IPAQ: the accelerometer recorded 3,072 min· wk^-1 ^being sedentary (with activity counts < 100) and study participants reported 3,092 min· wk^-1 ^sitting; this difference was not significant (*p *= 0.77). In the present study, however, time spent sitting was significantly underestimated by the IPAQ compared to the accelerometer by about 2 hours per day (*p *< 0.001).

The IPAQ may be more vulnerable than the PAR to the cognitive processing phenomenon known as forward telescoping [18]. This recall bias would occur if physical activity was recalled as taking place during the monitoring period but actually occurred in previous weeks [18]. The IPAQ provides a total of 35 exemplars of physical activities across the domains of activity, such as digging for vigorous yard work or raking for moderate yard work, so it may provide many opportunities for telescoping to occur. Such prompts have been shown to lead to this "forward telescoping" recall bias [18]. Unlike the PAR, the IPAQ required respondents to address a very thorough list of specific activities, which may possibly have led to over-reporting of activities during the prescribed 7-day monitoring period.

The IPAQ and PAR measures of physical activity are comparable on several dimensions. Both of the instruments used in this investigation measured physical activity during the past 7 days, and these were the days that the accelerometer was worn. Both instruments thoroughly assessed physical activity performed in all domains (occupation, housework, recreation, and travel-related). Both were administered by telephone and required a substantial amount of time to complete. However, the method that the IPAQ determined physical activity was through a series of standardized questions organized around the domains of physical activity. The PAR, on the other hand, used a semi-structured interview that prompted respondents to recall physical activities for specific segments of each day over the past week. The IPAQ defined vigorous physical activities as those "that make you breathe much harder than normal;" the PAR defined vigorous physical activity as the intensity one would associate with an activity between running and a brisk walk. Moderate physical activities on the IPAQ were defined as those "that make you breathe somewhat harder than normal;" moderate physical activity was defined as a brisk walk on the PAR. Thus, in an older population, as was used in this study, participants taking the IPAQ might classify physical activities of light intensity as being moderate in intensity. Those taking the PAR, however, would classify moderate activities with reference to an absolute criterion, i.e. taking a brisk walk.

While the IPAQ was developed for use for youth and adults aged 15 to 69, the PAR is generally used for adults, although no age limits have been specifically developed. The IPAQ required less training of the interviewer and was easier to administer, relying on a standard set of questions, although good instructions exist [11] to train and aid the administrator in conducting the PAR interview. In most cases the PAR is interviewer-administered, while a self-administered version of the IPAQ is available, making cost a potential consideration when using the PAR. Unlike the PAR, the IPAQ provided a measure of sedentary behavior which may be an important independent predictor of health outcome [25]. The IPAQ has been translated into several languages and so provides a standardized instrument for the measurement of physical activity internationally.

### Limitations

The present investigation had a number of methodological limitations. The study relied on a convenience sample of women diagnosed with breast cancer, participants were not able to complete both the IPAQ and the PAR, and they were also not randomly assigned to complete either the IPAQ or PAR. Although randomization was not possible, there were no differences in physical activity level, body mass index, age, percent non-Hispanic white, or education level, indicating that the two groups were comparable. While the methodological limits of the study must be recognized, we expect similar results are likely to be obtained in other samples using better methodology. Most of the samples in the multi-country IPAQ validation study were convenience samples [7], as well as many of the samples used in PAR validation studies [3]. The present validity correlation coefficients obtained on the IPAQ and measurement bias results are consistent with previous research [7,24].

The generalizability of these results may be limited to women in this region (Southern California) with an average age of 55 years, diagnosed with breast cancer, and enrolled in a clinical trial. Thus, the psychometric properties obtained in this sample may not generalize to women enrolled in other studies. Although study participants were diagnosed with breast cancer, most (79%) indicated that their physical activity was not limited in any way during the week that they wore the monitor. Of the 21% who reported that their physical activity was limited, most (50%) of the limitations were due to a cold, migraine, or sinus infection, 35% were due to musculoskeletal problems (knees, back, or foot), and 15% were disease-related (anemia, congestive heart failure, lung cancer, and lymphedema). These results suggest that most of the women, who on average were 4.7 years post-diagnosis, appeared to be in fair to good health.

Approximately 10% of the women were older than the targeted age range of the IPAQ, which ranges from 15 to 69 years. However, when women 70 years or older were eliminated from the analyses, the validity correlation coefficients were still significantly different from each other (.39 for the IPAQ vs. .73 for the PAR; *p *< 0.05). When outliers were eliminated from the analyses, the validity correlation coefficients remained exactly the same. Mean measurement bias of the IPAQ without outliers was +230 min/wk (compared to +257 min/wk with outliers) while the median measurement bias remained the same as did all indices of bias on the PAR. These analyses suggest that the validity and measurement bias findings were robust, whether including outliers or those older than 70 years.

The categorization of time spent in light, moderate, and vigorous physical activity according to the accelerometer was possibly more consistent with PAR than with IPAQ scores. Due to the method that intensity was defined on the PAR vs. the IPAQ (brisk walk vs. breathing harder than normal for moderate physical activity, for example), women taking the IPAQ might more likely assign light physical activities to those of moderate intensity. One way to account for this difference would be to investigate the use of age-adjusted scoring protocols for the IPAQ. Such use appears to be supported by post hoc correlational analyses stratified by age (55 years and younger vs. 56 years and older). Spearman validity correlation coefficients on the IPAQ for those aged 56 years and older (*N *= 39) were attenuated compared to those aged 55 years and younger (*N *= 57); similar findings were not observed on the PAR. Specifically, on the IPAQ, the correlations were 0.28 vs. 0.05, and 0.56 vs. 0.30, 0.42 vs. 0.32, when comparing younger vs. older women on moderate, vigorous, and total physical activity, respectively. Comparable correlations on the PAR were 0.49 vs. 0.84 and 0.62 vs. 0.84 for moderate and total physical activity in the younger vs. older group, respectively (no vigorous physical activity was recorded on the accelerometer among the older women who took the PAR). These results suggest that among older participants, the IPAQ may more often lead to misclassification of intensity level, be more inconsistent than the PAR with MET-based accelerometer categorization of intensity level, and that age-adjusted scoring of the IPAQ may be warranted.

An accelerometer was used to validate the self-report measures because it provided an objective measure of physical activity which was feasible and cost-effective. However, the accelerometer had a number of limitations. As noted previously, the accelerometer underestimated several sources of physical activity [23,26]. However, the technical reliability of the Actigraph indicates that it is very precise [27].

In the future, researchers using the IPAQ might consider assessing walking which is at least moderate in intensity. This would help distinguish between this potential source of over-reporting and that due to any forward telescoping. Reducing the number activity exemplars may reduce possible forward telescoping, but clarity of physical activity intensity terms may be compromised. Due to the problems of the accelerometer as the criterion, other more accurate "gold standards" might be considered in the validation of the physical activity self-reports, such as doubly labeled water methods combined with observational data.

## Conclusion

In summary, although the International Physical Activity Questionnaire provided a measure of sedentariness, was standardized for use internationally, and could be administered with limited training; its psychometric characteristics did not approach that of the 7-Day Physical Activity Recall. The magnitude of the differences in sensitivity and specificity between the PAR and long form of the IPAQ were impressive. The impact of these differences may be seen by considering a hypothetical case-control study in which 40% cases and 20% controls were physically inactive and the "true" odds ratio for physical inactivity and an outcome of interest was 2.7. If the PAR were the physical activity measure of choice, then the observed odds ratio would be 2.0. If the IPAQ were the measure of choice then the observed odds ratio would be a much lower 1.3. As the long form of the IPAQ is likely to be used in the same kinds of small- to medium-sized studies as the PAR — providing a thorough measure of physical activity performed over the past week — the choice of which instrument to use may have a significant impact on research results.

## Competing interests

The author(s) declare that they have no competing interests.

## Authors' contributions

JPP conceived the study. With the assistance of JPP, MJK designed the study, collected and analyzed the data, drafted the manuscript, and incorporated co-author input into manuscript revisions. JFS assisted in the design of the study and analysis and interpretation of the data. EAG and CLR assisted in the analysis and interpretation of the data. All co-authors provided critically important content-related revisions to the manuscript.
